# The Patent Medicine Curse

**Published:** 1904-07

**Authors:** 


### TEXAS MEDICAL JOURNAL.

AUSTIN, TEXAS.

A MONTHLY JOURNAL OF MEDICINE AND SURGERY.

EDITED AND PUBLISHED BY
F. E. DANIEL, M. D.
Mrs. F. E. DANIEL, Managing Editor.

Published Monthly at Austin, Texas. Subscription price $1.00 a year in advance.
Eastern Representative: John Guy Monihan, St. Paul Building, 220 Broadway, New York City.
Official organ of the West Texas Medical Association, the Houston District Medical Association, the Austin District Medical Society, the Brazos Valley Medical Association, the Galveston County Medical Society, and several others.

### THE PATENT MEDICINE CURSE.

One of the most hopeful signs of the times is an awakening of the lay press, and through it, the people, to some conception of the enormity of the evil of patent medicine, and a realization of the fact that, through this nefarious business the public is suffering in health and being victimized. Invalids, or fancied invalids, will believe the lies published about these cure-alls, and they are fleeced and poisoned outrageously. A lady asked me about a stuff that, according to the papers,—mostly church papers,—has cured more congressmen and generals than there are in the United States (and there are their pictures to *prove* it, if you don't believe it), and I advised her to let it alone. "Why," she said, "I see it *very* highly advised." Sure.

The consumption of patent medicines is one of the gravest and ever growing menaces to the public health. The gullible are being swindled to beat the band, and it is time that some notice were taken of it by the authorities. The medical press have preached it till it is threadbare; but no one sees or reads the preachment but doctors, and the evil is not reached. Nor have the medical men ever been able to get the lay press to take the matter up. Oh, no; it is grist to their mill, and many say, as did Jay Gould, "the public be damned." Hence it is a matter of much congratulation to see that the *Ladies Home Journal*, with a circulation of millions monthly, in the best homes, has fearlessly exposed and denounced the nefarious business, and warned the women (the women and children are the principal consumers and victims of these nostrums; the women *all* fancy themselves sick; many are, unfortunately), that they are being deceived, robbed and poisoned. The *Journal* gives the analysis (by the Massachusetts State Board of Health) of many of the well-known preparations, and shows that most of them contain alcohol, and the percentage runs from 20 to 47. It matters not that these fellows—the manufacturers and proprietors of these deadly frauds—are suing Curtis & Company, the *Home Journal* people, for millions for damages; the *Journal* has the authority of the Massachusetts Board of Health to fall back on, and they can easily prove their assertions. Anything that is true is not a libel. Of course they will push the suits, but Curtis & Company will beat them. They should have the support of the public. That of the majority of the medical press is assured; but the newspapers, and especially the church papers, whose revenue is largely derived from advertising these deadly deceits, will hands off or join the wolves. Some of the cure-alls for catarrh and for women's ailments contain as much alcohol as does good whisky; and the use of real good, straight whisky would not be half as injurious. It seems to me that they should all be classed as intoxicating liquors, and be subject to the liquor dealer's license, and prohibited in prohibition districts. There is something wrong somewhere, or they would be. It only emphasizes what the "Red Back" has insisted on for years: the necessity of a Commissioner of Public Health in the President's Cabinet. On proper representation to such an officer that alcohol is being sold as a patent medicine and the public poisoned by the indiscriminate use of secret remedies, so-called, he could prohibit it. The State laws are not enforced.

Perhaps the most directly injurious of these villainous compounds are those intended to produce abortion, the pennyroyal pills and cotton root teas. The church papers appear to make a specialty of this class of ads. It is particularly gratifying to note that the Postmaster General is taking steps to bar these and all other lying and injurious and fraudulent advertisements from the mails. All publications, lay or medical, carrying such advertisements will be excluded from the mail. He should go further. The law prohibits sending obscene matter through the mail, such as is calculated to corrupt the morals of the people. It is a penitentiary offense. If these abortion patent medicines and implements and devices intended clearly to produce abortion or prevent conception are not a bribe to girls, and a corruptor of morals, what are they? Oh that some Hercules would arise to clean out this Augean stable!

In the matter of the patent medicine exposure discussed above, it is with surprise and regret that our intelligent, interesting and highly esteemed contemporary the *Texas Medical Gazette* (Fort Worth), takes up a cudgel in defense of a notorious nostrum and ridicules the idea of its injuring anybody. It laughs to scorn the notion that 20 per cent of alcohol in such mixture (one-fifth) is detrimental to the health. It strikes me that whisky doctored with digitalis, even in small doses, taken every four hours, would scarcely improve one's health. The *Gazette* should condemn it on general principles, and specifically, if not on account of the whisky said to be sold under this disguise, because it is advertised in every newspaper and on every fence and blank wall, embellished with a giant size flaming picture of the "Doctor." The use of such nostrums indiscriminately is like shooting birdshot in the dark, and to make women believe it will cure them, irrespective of diagnosis, is a lie, a deceit, a crime.

---

A good example, one that should be followed by every county medical society in Texas, has been set by the Madison County Medical Society in publishing in their town papers a sensible, well written article on the prevention of typhoid and malarial fevers. It is addressed to the *people*, and is in plain language, easily understood by the dullest reader. The people are in total ignorance of the causes of disease, and of the dangers they are exposed to. They know nothing of sanitation and they must be taught. It is reasonable to suppose a man would drink, or permit his family to drink, the water from a sunk well if he knew it was contaminated by seepage from the surface or from a nearby privy sink, and contains the deadly typhoid germ? It is clearly the duty of the practicing physician to teach his clientele how to avoid being sick, and if the local papers will do as the *Meteor* has done, much good can be accomplished. I reproduce the paper, and suggest that every county society have it published or prepare and publish a similar address. All honor to Madison for taking the lead in this campaign of education.

---

The time for a second recurrence of yellow fever is at hand. Doctor, look out for *dengue!* Deal with your first case of *dengue*

as if it were real yellow fever, for it is! I am fully convinced that dengue is modified yellow fever, and although the first cases are usually mild and seldom fatal, the virulence of the poison increases as it spreads until we have fatal cases with all the symptoms strongly marked, when, presto! an epidemic of yellow fever is on! The close resemblance of dengue to yellow fever has long been noted, and it being so seldom fatal, and it nearly always precedes an outbreak of yellow fever, has puzzled the physicians of the South for fifty years. The view above expressed that they are identical, differing only in degree, will explain much that has seemed inconsistent and contradictory. A physician in Beyreut, Syria, has recently discovered and published the fact that dengue is, like yellow fever, transmitted by a mosquito. He does not name the species, but if true, it is doubtless the *steg. fas.* This discovery strengthens the belief that dengue is "attenuated" yellow fever. Look out for the wolf in sheep's clothing. Squelch the first cases and abolish quarantine.

---

AND NOW the "glorious fourth" is past, the voice of the festive tetanus bacillus will be heard in the land. The pretty little toy pistol that papa bought for Johnnie with its loud little dynamite cartridges with which to scare sister just for fun, and exasperate the neighbors, will be heard from. It has a record of 406 fatal cases of lockjaw to its credit for last fourth of July. Let us see how, like the little busy bee, it improves each shining hour, each recurring fourth, to mend its record. Will the people never learn wisdom?

---

SECRETARY IRA C. CHASE of the State Medical Association of Texas, is realizing the hopes and expectations of his friends. He is making a most efficient secretary, bringing to the office, as he does, intelligence and zeal. The organization is growing, and at the Houston meeting next April we count on 3000 active members.

